# The many faces of HFPEF: insights from the CMR substudy of DIAMOND-HFPEF

**DOI:** 10.1186/1532-429X-17-S1-P356

**Published:** 2015-02-03

**Authors:** Prathap Kanagala, Adrian S Cheng, Anvesha Singh, Jamal N Khan, Sheraz A Nazir, Iain B Squire, Leong L Ng, Gerry P McCann

**Affiliations:** 1 Cardiovascular sciences, NIHR Cardiovascular BRU, University of Leicester, Leicester, UK; 2 Kettering General Hospital, Kettering, UK

## Background

Heart failure with preserved ejection fraction (HFPEF) carries significant mortality and morbidity. This population is characterized by marked heterogeneity. To date, this population has been poorly phenotyped and it remains unclear what proportion may have evidence of myocardial infarction (MI), undiagnosed cardiomyopathies or phenocopies of heart failure. At present, echocardiography remains the primary modality for diagnosis and inclusion into randomized controlled trials but can be challenging due to the high prevalence of lung disease, obesity and frailty. We sought to evaluate the impact of adding cardiac magnetic resonance imaging (CMR) to the diagnostic pathway.

## Methods

CMR was offered as part of DIAMOND-HFPEF (Developing Imaging And plasMa biOmarkers iN Describing Heart Failure with Preserved Ejection Fraction): a prospective, observational, cohort study. Inclusion criteria were: clinical features of HF and left ventricular ejection fraction (LVEF) > 50% as per echocardiography. Exclusion criteria were: suspected or confirmed cardiomyopathy, pericardial constriction, inability to consent, non-cardiovascular life expectancy < 6 months, myocardial infarction in the preceding 6 months, severe valve disease, severe obstructive pulmonary disease (or FEV_1_ < 30% or FVC < 50% predicted) and estimated glomerular filtration rate < 30 ml/min/m^2^. 138 eligible subjects underwent 3 T CMR (Siemens Skyra) according to a standardized protocol including cine, first-pass perfusion and late gadolinium imaging. The clinical CMR reports were made available to patients' responsible physicians.

## Results

**Figure 1:**
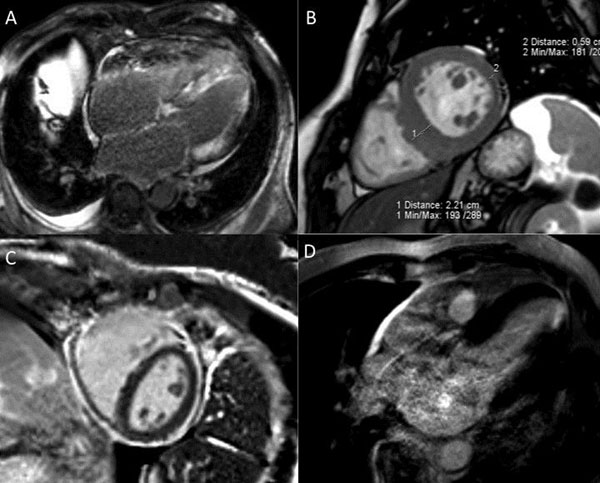
**Examples of new diagnoses based on CMR.** A) Late gadolinium imaging illustrating the hallmarks of cardiac amyloid - diffuse enhancement of the LV and atrial myocardium, dilated atria and pleural & pericardial effusions B) end-diastolic steady state free precession image of a patient with marked asymmetrical septal hypertrophy C) Late gadolinium imaging demonstrating thickened pericardium with contrast enhancement in a case of pericardial constriction D) Late gadolinium imaging demonstrating a small sub-endocardial apical lateral myocardial infarct of 50%-75% transmurality

Baseline clinical and CMR data are summarized in Table 1. Qualitative analyses revealed new diagnoses of (see Figure 1): ‘silent' myocardial infarction (n = 13), hypertrophic cardiomyopathy (definite [n = 4], probable [3], possible [n =1]), pericardial constriction (n = 5) and amyloid (n = 2). Perfusion imaging revealed reversible inducible ischaemia in 27 patients, of which 11 were likely to be secondary to microvascular dysfunction. Late gadolinium imaging revealed mid-wall focal fibrosis in 33 patients and insertion point fibrosis in 24 patients.

**Table 1:** Baseline Clinical and CMR Characteristics

Age (years) 74 ± 10	BMI (kg/m2) 33 ± 8
Male (n, %) 73 (54)	BMI > 35 kg/m2 (n,%) 46 (34)

Sinus Rhythm (n, %) 90 (67)	NYHA class (n, %)

Atrial Fibrillation/Flutter (n, %) 44 (33)	I: 38 (16)

Heart Rate (bpm) 70	II: 51 (40)

Systolic BP (mmHg) 143	III: 41 (31)

Diastolic BP (mmHg) 73	IV: 4 (3)

Diabetes (n, %) 71 (53)	BNP (pg/ml); (median, IQR) 182.3 (166.2, 286.0)

Hypertension (n, %) 125 (93)	Serum Creatinine (µmol/l) 102 ± 34

Coronary Artery Disease (n, %) 27 (20)	LVEF (%) 57 ± 10

Angina (n, %) 26 (19)	LVEDVI (ml/m2) 76 ± 26

COPD/Asthma (n, %) 20/6 (15/4)	LVESVI (ml/m2) 33 ± 14

bpm = Beats per minute ; BP = Blood Pressure ; COPD = Chronic Obstructive Pulmonary Disease ; BMI = Body Mass Index ; NYHA = New York Heart Association ; BNP = Brain Natriuretic Peptide ; IQR = Inter-Quartile Range ; LVEF = Left Ventricular Ejection Fraction ; LVEDVI = Left Ventricular End Diastolic Volume Index ; LVESVI = Left Ventricular End Systolic Volume Index

## Conclusions

CMR provides unique insights into etiology, enabling disease-specific management and the potential for tailored therapies to improve outcomes in HFPEF. Furthermore, the frequent finding of HFPEF ‘phenocopies' such as hypertrophic cardiomyopathy and amyloid in our cohort may in part explain the limited efficacy of generic medical therapies in numerous randomized controlled studies.

## Funding

DIAMOND-HFPEF is funded by the NIHR Leicester Cardiovascular Biomedical Research Unit.

